# Protein condensation diseases: therapeutic opportunities

**DOI:** 10.1038/s41467-022-32940-7

**Published:** 2022-09-22

**Authors:** Michele Vendruscolo, Monika Fuxreiter

**Affiliations:** 1 Centre for Misfolding Diseases, Yusuf Hamied Department of Chemistry, University of Cambridge, Cambridge, UK; 2 Department of Biomedical Sciences, University of Padova, Padova, Italy

**Keywords:** Drug discovery, Chemical biology, Protein folding

## Abstract

Condensed states of proteins, including liquid-like membraneless organelles and solid-like aggregates, contribute in fundamental ways to the organisation and function of the cell. Perturbations of these states can lead to a variety of diseases through mechanisms that we are now beginning to understand. We define protein condensation diseases as conditions caused by the disruption of the normal behaviour of the condensed states of proteins. We analyze the problem of the identification of targets for pharmacological interventions for these diseases and explore opportunities for the regulation of the formation and organisation of aberrant condensed states of proteins.

## Abstract (web summary)

In this review, the authors define protein condensation diseases as conditions caused by aberrant liquid-like or solid-like states of proteins, and describe opportunities for therapeutic interventions to restore the normal phase behaviour of proteins. The review accompanies the related collection of articles published in Nature Communications focusing on possible therapeutic approaches involving liquid-liquid phase separation.

## Introduction

By folding into their native states, proteins perform myriad molecular functions that are essential for the maintenance of cellular homoeostasis^1^. The phenomenon of protein folding is a prominent example of the ability of biological systems to self-assemble by bringing together reactive groups in complex arrangements that enable sophisticated biochemical functions.

**Fig. 1:**
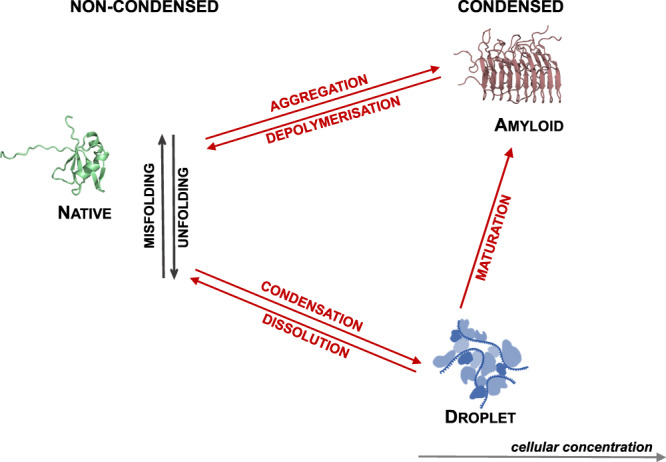
Protein condensation diseases are conditions caused by the aberrant conversion of proteins between the native, amyloid and droplet states. Under cellular conditions, many proteins, in addition to the native state, can populate two condensed states, the liquid-like droplet state and the solid-like amyloid state^4,10^. Protein condensation diseases are the consequence of the failure of the protein homoeostasis system to regulate the balance between the different protein states (Fig. 2). A list of currently known protein condensation diseases is provided in Table 1.

In recent years, it has also emerged that the ability of proteins to organise themselves into functional forms extends beyond native states. Numerous proteins have been shown to undergo a liquid-liquid phase separation process leading to the formation of membraneless organelles, which are complex biomolecular assemblies resembling a dense liquid-like state^2,3^, also referred to as the droplet state^4^. Furthermore, many proteins can also form a highly ordered solid-like state, known as the amyloid state^5^, which in certain cases can be functional^6,7^. Since in the cell most proteins are typically expressed at concentrations at which they can form condensed states^8,9^, the droplet and amyloid states could be considered as fundamental states of proteins along with the native state^4^ (Fig. 1).

Proteins in condensed states can perform a wide range of biological functions by increasing the efficiency of cellular processes and by reducing biological noise^10,11^. The increase in the local concentrations of different cellular components in condensed states accelerates enzymatic reactions, such as in the cases of the premelanosome protein (Pmel17) in melanin synthesis^12^ and of cyclic GMP-AMP synthase (cGAS) in innate immune signalling^13^. Liquid–liquid phase separation can amplify signals by low-affinity effectors and ligands by facilitating the formation of signalling clusters, such as in T cell receptors^14^ or Wnt signalling^15^. Droplets can serve as non-membrane bound cellular compartments, such as the nucleolus^16^ or facilitate their formation through nucleation of polymerisation reactions, such as microtubulin for centrosome formation^17^. The assembly and disassembly of condensates promote morphological changes in developmental processes, such as the pattern specification process^3^. Condensates may orchestrate components of cellular pathways, such as in the cases of p53-binding protein 1 (53BP1) droplets, which concentrate components for DNA repair^18^ or of heterochromatin protein 1 (HP1) droplets, which induce gene silencing^19^. Furthermore, an increasing number of cellular processes have been associated with solid-like scaffolds^6,7^. In particular, signalling complexes in the innate immune system, such as inflammasomes, faddosomes, myddosomes often form solid-like condensates^20,21^ to recruit downstream signalling components.

In this work, we first characterise protein condensation diseases as disorders caused by aberrant liquid- or solid-like states of proteins. We then address the problem of identifying possible targets for drug discovery in order to restore the normal phase behaviour or proteins.

### Regulation of protein condensation by the protein homoeostasis system

**Fig. 2:**
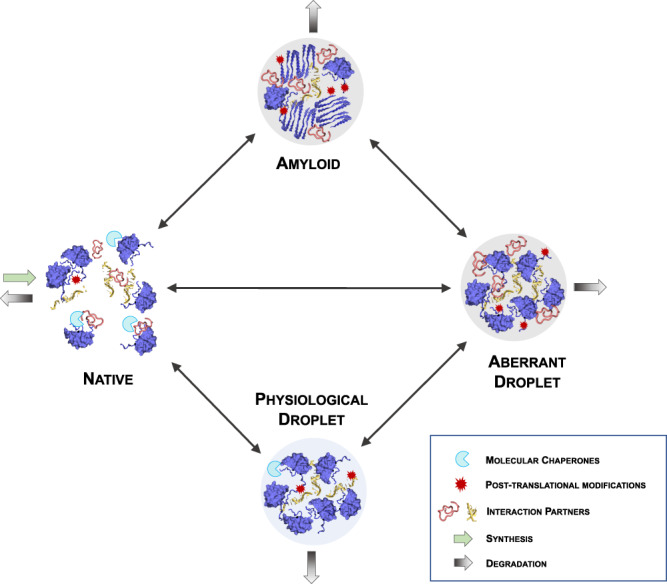
Protein condensation and protein homeostasis. The protein homoeostasis system regulates the formation, clearance, composition, interactions, localisation and biophysical properties of protein condensates^146,147^. Although the complete mapping of the protein homoeostasis system that controls protein condensates is still far from complete, several examples have already been identified. The formation and dissolution of the droplet state are regulated by post-translational modifications^23,24^ and the availability of interaction partners^25^. The re-localisation within a cell of solid-like condensates may revert them to the liquid-like state by making available suitable interaction partners^34,117^. Molecular chaperones may interfere with misfolded protein intermediates and inhibit the formation of the amyloid state either from the native state through the deposition pathway or from the droplet state through the condensation pathway^27^. Autophagy contributes to stress granule clearance^29^, and the liquid-liquid phase separation of p62 with its ubiquitinated substrates may lead to autophagosome formation^30^.

The balance between the condensed states and the native state of proteins must be highly regulated for optimal functions. The protein homoeostasis system controls in multiple ways the process of protein condensation, including the reversible formation of the droplet state from the native state, its irreversible maturation to the amyloid state, as well as the irreversible aggregation of the native state to the amyloid state^5,22^ (Fig. 2).

The assembly and dissolution of the droplet state in response to specific cellular conditions is often regulated through post-translational modifications^23,24^ (Fig. 2). The protein kinase Sky1, for example, controls stress granule disassembly through the phosphorylation of the nucleocytoplasmic mRNA shuttling protein Npl3^25^. Alternative mechanisms for stress granule clearance are provided by molecular chaperones^26^, in particular in the case of aberrant condensates containing misfolded proteins^27^ (Fig. 2). The two mechanisms are linked, as Sky1 overexpression can compensate chaperone defects in stress granule disassembly pathways^25^. The level of ubiquitination also controls stress granule formation, for example, depletion of the deubiquitylases USP5 and USP13 resulted in accelerated stress granule assembly and delayed the return to normal conditions^28^.

Stress granule clearance in mammalian cells can be also reduced by inhibition of autophagy, or by impairment of valosin-containing protein (VCP, the human ortholog of CDC48), which plays a critical role in protein quality control^29^. Droplet clearance by autophagy involves liquid-liquid phase separation of the ubiquitinated substrate and the ubiquitin-binding protein p62^30^. p62 condensates are further regulated by the death-domain-associated protein DAXX^31^ and contribute to the oxidative stress response mediated by the transcription factor Nrf2^32^. p62 condensates and their interactions with the nuclear receptor Nur77 are also critical for the removal of damaged mitochondria^33^. Interactions with nuclear transport receptors regulate cellular localisation and condensate assembly, as it was shown for the TAR DNA-binding protein 43 (TDP-43) and the RNA-binding protein senataxin (SETX) in spinal cord motor neurons^34^.

### Protein condensation diseases

As a counterpart to the wide range of the cellular processes described above, it is becoming increasingly clear that failures in the regulation of condensed states may lead to dysfunctional protein assemblies that could be involved in a range of pathological processes^22,35,36^.

Numerous pathological conditions have been mechanistically linked to the formation of aberrant liquid-like^22,35,37,38^ and solid-like^5,39^ condensates (Table 1). It is thus becoming increasingly clear that aberrant protein condensation likely has a causative nature in a wide range of human diseases. These pathologies, which can be collectively defined as protein condensation diseases, originate in alterations of the physiological states of proteins (Fig. 1), due to the failure of regulating the formation, clearance, composition, interactions and localisation of protein condensates (Fig. 2). In the following, we discuss examples of protein condensation diseases as conditions caused by the disruption of the normal behaviour of the condensed states of proteins.

**Table 1:** Examples of currently known diseases associated with aberrant protein condensation

Disease	Protein	Missense mutations	Type	Classification
Primary immune-deficiency syndromes^122^	IRAK4	R12C, R20W	*LoF*	Amyloid to native
Primary immune-deficiency syndromes^122^	MYD88	S34Y, S98C	*LoF*	
Autoimmune lymphoproliferative syndrome^123^	FAS	R250Q, A257D, D260Y, T270K, E272K	*LoF*	
Amyotrophic lateral sclerosis^52,102,124^	FUS	G156E, G187S, G225V, G230C, G399V, P525L, R244C, R216C, R521G	*LoF*	Droplet to amyloid
Amyotrophic lateral sclerosis^50,125,126^	TDP-43	A321V, G298S, M337V, A315T	*LoF*	
Amyotrophic lateral sclerosis^101,127–130^	HNRNPA1	D262V, D214V, P298L, D290V	*LoF*	
Amyotrophic lateral sclerosis^53^	TIA1	P362L, A381T, E384K	*LoF*	
Amyotrophic lateral sclerosis^114^	UBQLN2	P506A, P506S, P506T, P497H, P497S,	*GoF*	
Amyotrophic lateral sclerosis^131^	CHCHD10	S59L	*LoF*	
Frontotemporal dementia^131^	CHCHD10	S59L	*LoF*	
Alzheimer’s disease^132^	TAU	P301L, P301S, A152T	*LoF*	
Progranulin deficiency^54^	TDP-43		*LoF*	
Limb-girdle muscular dystrophy 1G^133^	HNRNPDL	D378H, D378N	*LoF*	
Huntington disease^134^	HTT	43QP	*LoF*	
Distal hereditary motor neuronopathy^72^	HSPB3	R116P	*LoF*	
Cervix cancer^77^	UTX	S781Y	*LoF*	
Autosomal-dominant distal myopathy^135^	MATR3	S85C	*LoF*	Droplet to native
Prostate cancer^46^	SPOP	F133V, W131G	*LoF*	
Rett syndrome^40^	MECP2	P225R, R306C, P322L	*LoF*	
Atopic dermatitis^42^	FLG	Tail-deficient mutants	*LoF*	
Mental retardation autosomal dominant 5	SYNGAP1	T1305A	*LoF*	
Inclusion of body myopathy with early-onset Paget’s disease^29^	VCP	A232E	*LoF*	
Frontotemporal dementia^29^	VCP	A232E	*LoF*	
Amyotrophic lateral sclerosis 14 without FTD^29^	VCP	R155H	*LoF*	
Paget’s disease^47^	SQSTM1	M404V	*LoF*	
Dementia^47^	SQSTM1	M404V	*LoF*	
Usher syndrome^136^	MYO7A	K2021R, L2186P, G2187D	*LoF*	
Skin cancer^136^	MYO7B	E1288K	*LoF*	
Amyotrophic lateral sclerosis^114^	UBQLN2	T487I, P497L	*GoF*	Native to droplet
Amyotrophic lateral sclerosis^34^	SETX	L389S, R2136H	*GoF*	
Central nervous system cancer^119^	DDX3X	T275M, G302V, G325E, M370R	*GoF*	
Dilated cardiomyopathy^137^	RBM20	R636S	*GoF*	
Inherited taupathy^43^	TAU	P301L	*GoF*	
Respiratory syncytial virus infection^45^	M2-1		*GoF*	
Multisystem proteinopathy^47^	TIA1	N357S	*GoF*	
Noonan syndrome, Leopard syndrome^108^	PTPN11	G464A	*GoF*	
Juvenile myelomonocytic leukaemia^108^	PTPN11	D61G, E76A, E76K, Q506P	*GoF*	
Liver cancer^108^	PTPN11	Y279C	*GoF*	
Lung cancer^138^	KEAP1	R320Q	*LoF*	
Skin cancer^138^	KEAP1	R470C	*LoF*	

Disease-associated missense mutations shown to alter protein condensation are listed. Loss-of-function (LoF) and gain-of-function (GoF) mutations are classified based on the original studies. Misfolding diseases classified as native to amyloid are reviewed elsewhere^39^, and diseases that could be classified as amyloid to droplet are not currently known.

Perturbations that induce the disassembly of liquid-like condensates may compromise their physiological functions. For example, with the condensation of methyl CpG binding protein 2 (MeCP2) being critical for heterochromatin assembly, it has been reported that mutations that disrupt this process lead to transcriptional dysregulation in Rett syndrome^40^ (Table 1). Mutations of MeCP2 associated with Rett syndrome can also impair the formation of the RNA-binding fox-1 (Rbfox) condensates, compromising their splicing functions^41^. It has also been shown that the failure in the formation of keratophyalin granules compromises skin defence mechanisms in atopic dermatitis^42^.

Conversely, the droplet state can potentially concentrate harmful conformations or pathogenic material. For example, liquid-like droplets can stabilise cytotoxic assemblies of tau, which promote tau aggregation in Alzheimer’s disease^43^ (Table 1). It has also been reported that viral replication can take place in virus-induced inclusion bodies^44^, as observed in respiratory syncytial viral infections^45^.

More generally, shifting the phase boundary either towards the formation of condensates or towards their disassembly can have pathological consequences. Cancer-causing mutations in the speckle-type POZ protein (SPOP), by reducing its tendency to phase separate, lead to a failure in its co-localisation with DAXX, thus dysregulating ubiquitin-dependent protein homoeostasis^46^ (Table 1). In contrast, mutations in p62, by disturbing stress granule clearance, lead to multisystem proteinopathy and Paget’s disease^47^. A loss of liquid-like properties of the condensates of A-kinase anchoring protein (AKAP95) may cause tumorigenesis by compromising splicing functions^48^.

A wide range of disorders is caused by the shifting of the droplet state towards the amyloid state^49^. The irreversible maturation of granules of RNA-binding proteins, including TDP-43^50^, heterogeneous nuclear ribonucleoprotein A1 (hnRNPA1)^51^, fused in sarcoma (FUS)^52^ and T-cell intracellular antigen 1 (TIA)^53^, can results in loss of function, as for example in amyotrophic lateral sclerosis (ALS) and frontotemporal dementia (FTD). The conversion of the droplet state into the amyloid state may lead to loss of function by amyloid fibril formation, as well as the formation of promiscuous intermediates causing cytotoxicity^53^. Protein aggregation may be induced by a deficiency of an interaction partner, such as in the case of progranulin, whose down-regulation contributes to microglial toxicity of TDP-43^54^. Droplet maturation, however, may also be required for physiological functions. For example, the innate immune mechanism involving the virus-induced inflammasome formed by NOD-like receptor family pyrin domain containing 6 (NLRP6) undergoes solidification after the recruitment of apoptosis-associated speck-like protein (ASC) for downstream activation^55^. Likewise, an acquisition of solid-like behaviour of the condensates of the active-zone scaffold proteins SYD-2 and ELKS-1 is required for synaptic functions^56^.

Perturbing the interplay between membrane-bound organelles and condensates may lead to additional disease mechanisms^57–59^. Ribonucleoprotein granule biogenesis is modulated by the contact sites with the endoplasmic reticulum (ER), which regulate the fusion and fission of processing bodies (P-bodies) and stress granules^60^. This process couples the ER translational capacity with the generation of membraneless organelles. In a similar vein, the ER forms a compartment with TIS granules, which through interactions between 3’ untranslated RNA regions modulates the expression of membrane proteins^61^. In addition, interactions with ER membranes affect the size of Whi3 membraneless organelles, thereby limiting the local concentration increase on the ER surface^62^. Via modulating protein concentrations, ER-linked STING protein condensates influence innate immunity responses^63^. Although growing evidence demonstrates the biological importance of condensate-organelle communications, only a few disease-associated mutations have been directly linked to this process. As annexin A11 enhances RNA transport in neurons by tethering RNP granule cargos to lysosomes^64^, ALS-associated mutations of annexin A11 disrupt its interactions with lysosomes and impair its adaptor function^64^.

### Classification of protein condensation diseases

To identify links between condensate-forming proteins (Supplementary data set: Table S1) and human disease, we searched for pathologies associated with genes encoding these proteins. Our analysis indicates that up to a third of human diseases can be associated with genes that encode condensate-forming proteins (Supplementary data set: Table S2), and that missense mutations in these genes accumulate in the droplet-promoting regions of the corresponding proteins (Supplementary data set: Table S3). The aim of these rankings is to help future studies identify diseases in which protein condensation has a causative nature, and corresponding possible targets for pharmacological intervention (Tables S1, S2 and S3).

The top disease categories based on gene-disease associations (Supplementary data set: Table S2) include abnormal tissue morphology changes, such as breast, liver, colorectal, prostate, lung tumours, stomach carcinoma, glioblastoma. These aberrant condensates lead to dysregulation of gene-expression programs^46^, cell division or failure of DNA repair processes^18^. The liquid-like properties of droplets can also promote morphological changes by concentrating selected components for cancer development and metastasis^65^. Top-ranking protein condensation diseases also include nervous system disorders, such as schizophrenia, bipolar and autistic disorders, depression, epilepsy, as well as Alzheimer’s and Parkinson’s diseases. Most of these neurological disorders are associated with genes encoding proteins forming synaptic condensates^66^. As synaptic plasticity requires in many cases a liquid-liquid phase separation of synaptic proteins^67^, aberrant protein condensation was shown to compromise synaptic functions^66,68^. In addition, according to our analysis, aberrant condensates of cytoskeletal^14^ and signalling proteins^69^ are likely to contribute to these neurological disorders. We also identified cardiovascular protein condensation diseases, such as myocardial ischaemia, atrial fibrillation, myocardial failure, atherosclerosis and cardiomyopathy (Supplementary data set: Table S2). Troponin, a key marker of myocardial infarction, and proteins controlling the circadian clock were associated with nuclear condensates^70,71^. Aberrant phase separation can perturb nuclear functions, as demonstrated for small heat shock proteins associated with cardiac myopathy^72^, and contribute to different muscular dystrophies, as illustrated by the case of the membraneless compartmentalisation of Z-disk proteins, which is essential for myofibrillogenesis^73^. We also identified digestive system disorders (Supplementary data set: Table S2), such as liver cirrhosis, hepatitis, alcoholic intoxication, that involve genes encoding condensate-forming proteins. These include cytosolic glutathione-S-transferases, the urea cycle enzyme carbamoyl phosphate synthase I, several enzymes involved in amino acid metabolism, and cholesterol transport, as components of cellular bodies formed in response to stress^74,75^. Aberrant protein condensation of metabolic enzymes is associated in our analysis with a wide range of disorders, including diabetes mellitus and metabolic syndrome (Supplementary data set: Table S2). Energy stress was shown to modulate localisation and condensation of glycolytic enzymes^76^. We also identified immune system disorders (polyarthritis, asthma) and viral infections (influenza) associated with genes encoding PYD and CARD domain-containing proteins, the condensation of which is required for innate immune signalling^13,55^ (Supplementary data set: Table S2).

Next, based on the analysis of disease-associated missense variants, we identified over 600 disorders that can be classified as protein condensation diseases (Supplementary data set: Table S3), as most contributing mutations are in droplet-promoting regions of experimentally identified condensate-forming proteins (Supplementary data set: Table S1). This classification included rare multisystem disorders such as the Kabuki^77^, Werner and Rubinstein-Taybi syndromes, which have a high fraction of droplet-associated mutations and involve various biological pathways (Supplementary data set: Table S4). Thus, we systematically analysed the genes associated with 3178 orphan diseases from the Orphanet database (https://www.orpha.net) and found that over 2168 orphan diseases (i.e. over two-thirds) have a considerable contribution from genes encoding droplet-forming proteins (Supplementary data set: Table S2). Furthermore, we identified 140 rare disorders for which most missense mutations are associated with known droplet-forming proteins (Supplementary data set: Table S3). This analysis indicates that many orphan diseases are likely to be associated with protein condensation, which can offer mechanisms for targeting these pathologies. This observation can for example be exploited for screening compound libraries for these disorders, including by using fluorescent markers of components forming aberrant condensates.

### Interactions within protein condensates in health and disease

We are only beginning to understand the molecular forces that drive liquid-liquid phase separation by finely tuning the balance between the native and condensed states in the cellular environment^4,78–80^. The formation of the liquid-like condensed state has been initially associated with the presence of disordered regions^81^ and of prion-like domains^82^ in RNA-binding proteins. However, increasing numbers of structured proteins, ranging from metabolic enzymes^77^ to signalling complexes^83^, have also been observed to undergo liquid-liquid phase separation. These observations suggest that the inter-molecular interactions driving condensate formation could have a more generic nature and be more widespread in the proteome^8,9,84^.

According to our current understanding of the protein condensation process, liquid-like condensates are stabilised and regulated by disordered interactions^4,78–80,85^, while the formation of solid-like aggregates requires the self-assembly of inter-backbone hydrogen-bonding networks into highly ordered amyloid structures^86^. The process of liquid–liquid phase separation can be driven by a wide range of sequence motifs including electrostatic (π–π^87^ and charge–π^88^) and hydrophobic^89^ interactions. Organisation of such non-canonical motifs into patterns, such as those of aromatic and charged residues, was observed to enable phase separation^88,90^ Perturbing interaction patterns modulates the conformational propensity of a protein sequence^91^, which can shift the droplet state to the native state^88^. Along these lines, linker regions contribute to phase separation by influencing the number of accessing binding, such as in the case of the adaptor protein Nck^92^.

The multivalent interactions driving liquid–liquid phase separation exhibit strong dependence on the cellular context^79,93^, including the pH^94^ and salt concentration^78^. Cellular localisation and concentration of interaction partners, including RNA, are critical for promoting the formation and controlling the properties of condensates^95,96^. Together with hydrophobic interactions, aromatic interactions are important under high salt conditions, while electrostatic interactions dominate the process under low salt conditions^78^. Post-translational modifications and allosteric effects of the flanking regions can provide a further layer of regulation to switch the motifs on and off^19,68^. For example, phosphorylation regulates the formation of FMRP and caprin-1 condensates to control mRNA deadenylation^97^, dual-specificity kinases are important regulators of condensate homoeostasis^23,24^, and histone H1 acetylation antagonises chromatin phase separation^98^.

**Fig. 3:**
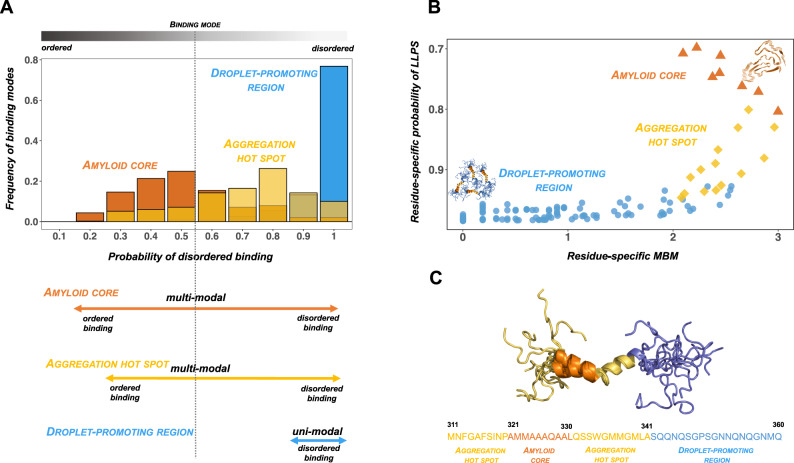
Inter-molecular interactions within protein condensates in health and disease. **A** Interaction modes of residues in the prion-like domain of TDP-43 vary between disordered and ordered modes. The interaction motifs that promote the formation of the condensed states of this protein are influenced by their flanking regions. The TDP-43 amyloid-core region (residues 321–330, orange) and the flanking aggregation hot-spots (residues 312–320 and 331–342, yellow) sample both ordered and disordered interactions (multi-modal binding). In contrast, most residues outside these regions are droplet-promoting (residues 262–311 and 343–414, blue), which sample mostly disordered interactions (unimodal binding). **B** The droplet landscape of TDP-43 prion-like domain illustrates the conversion between droplet and amyloid states. The likelihood of aggregation within droplets depends on two features^99^, the residue-specific multiplicity of binding modes (MBM) and the probability of undergoing liquid–liquid phase separation (LLPS). The multiplicity of binding modes characterises the ability of sampling both disordered interactions, which bias towards the droplet state (blue, based on PDB: 2N3X^148^), and ordered interactions, which bias towards the amyloid state (PDB:7KWZ^149^, orange). Both properties can be predicted from the sequence using the FuzDrop method (https://fuzdrop.bio.unipd.it)^8^. Droplet-promoting regions (blue circles) have a low multiplicity of binding modes in contrast to the amyloid core (orange triangles) and aggregation hot-spots (yellow diamonds), which exhibit high multiplicity of binding modes (large *y* values)^99^. **C** The sequence of the amyloidogenic region of TDP-43 (residues 311–360) is shown corresponding to the solution structure (PDB: 2N3X). The amyloid core is shown by orange, the aggregation hot-spot by yellow and the flanking residues by blue. The liquid–liquid phase separation of the prion-like domain of TDP-43 depends on the presence of an α-helical structural element^125^.

Taken together, these observations suggest that the formation of the droplet state is mediated by disordered interactions, while that of the solid-like amyloid state by ordered interactions^99^ (Fig. 3). Neurogenerative diseases are thus in many cases associated with mutations that increase the multiplicity of binding modes by promoting interactions that promote both the droplet and amyloid states. Thus, regions that can sample both types of interactions can drive amyloid formation within condensates^100^. Charge–π interactions, for example, can lead to reversible amyloid formation, while the mutation of charged residues into hydrophobic ones can stabilise the amyloid state^101^. Familial mutations associated with neurodegenerative disorders may expand the repertoire of binding modes of a protein, such as in the case of FUS G156E^102^, enabling a gradual shift towards more ordered configurations of condensates. Indeed, ALS-associated and non-ALS-associated mutations of RNA-binding proteins can be distinguished on the basis of the sequence-based calculation of physico-chemical properties of proteins, including droplet and aggregate propensities, and diversity of interaction modes^99^.

The nature of the inter-molecular interactions stabilising the droplet and amyloid states can be illustrated using the example of the prion-like domain of TDP-43 (Fig. 3A). Depending on the sequences of flanking regions^103^, the residues of the amyloid core and the flanking regions exhibit a multiplicity of binding modes (Fig. 3A). This property turns these residues into aggregation hot-spots that induce the conversions of the liquid-like into the solid-like state. In contrast, residues that promote droplet formation exhibit unimodal interactions and mostly sample disordered interactions (Fig. 3A). Thus the multiplicity of binding modes is a key feature to characterise the likelihood of conversion between the droplet and amyloid states, together with the residue-specific probability of undergoing liquid–liquid phase separation, as represented by droplet landscapes^99^ (Fig. 3B).

### Therapeutic opportunities for protein condensation diseases

**Fig. 4:**
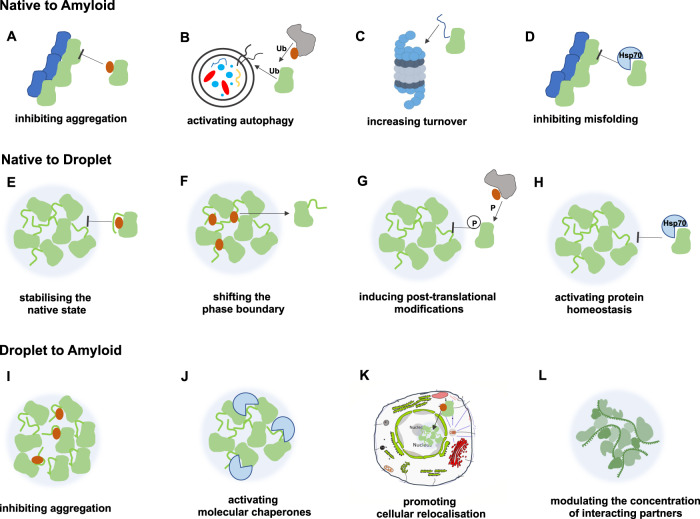
Examples of therapeutic opportunities for protein condensation diseases. Small molecules and antibodies are shown by brown circles. Candidate drugs can: (i) directly bind short sequence motifs that drive the formation of condensed states or stabilise them (**A**, **F**, **I**), (ii) interfere with the regulation of the assembly and disassembly of condensed states (**G**), (iii) modulate the stability of the native state (**D**, **E**, **H**, **J**), (iv) modify the concentration of a protein or its partners via inhibiting synthesis or inducing degradation (**B**, **C**, **L**), or (v) re-localise the protein itself (**K**). Examples of currently investigated approaches are listed in Table 2.

The observation that condensate-forming proteins appear to be ubiquitous in human disease opens the way to the development of therapeutic strategies capable of modulating their condensation behaviour and restore their physiological states (Table 2 and Fig. 4).

**Table 2:** Examples of therapeutic opportunities for protein condensation diseases

Protein state perturbation	Therapeutic opportunities	Mechanism of action	Examples
Native to amyloid	Antibodies against protein aggregation	Removal of protein aggregates Inhibition of the protein aggregation process	Aβ^112,113^
	Small molecules against protein aggregation	Stabilisation of the native state Inhibition of the protein aggregation process	TTR^111^, Aβ^109,110^
	Small molecules promoting protein degradation	Activation of autophagy and of the ubiquitin–proteasome system to remove aggregating proteins and protein aggregates	mTOR^139^, PKA^140^
	Small molecules inhibiting protein synthesis	Inhibition of enzymes required for the production of aggregating proteins	Aβ^141^
	Small molecules promoting the unfolded protein response	Activation of the unfolded protein response to remove aggregating proteins and protein aggregates, or to inhibit the formation of protein aggregates	PERK^142^
	Molecular chaperones against protein aggregation	Activation of the protein homoeostasis system to remove protein aggregates or inhibit their formation	Hsp70^143^
Native to droplet	Small molecules against protein liquid-liquid phase separation	Stabilisation of the native state Perturbation of protein interactions within droplets	SHP2^108^, M2-1^45^, tau^106^
	Small molecules promoting protein degradation	Activation of autophagy to remove droplet-forming proteins	tau^106^
	Small molecules regulating post-translational modifications	Activation of the protein homeostasis system to inhibit droplet formation	FUS^144^
	Molecular chaperones against protein liquid-liquid phase separation	Activation of the protein homeostasis system to inhibit droplet formation	FUS^144^
Droplet to native	Small molecules regulating post-translational modifications	Prevention of droplet dissolution	DYRK3^23^
Droplet to amyloid	Small molecules against protein aggregation	Inhibition of the protein aggregation process	α-synuclein^145^
	Molecular chaperones against protein aggregation	Stabilisation of folded protein domains within droplets	FUS^116^
	Molecular chaperones regulating cellular localisation	Re-localisation of nuclear RNA-binding proteins	FUS, hnRNPA1, hnRNPA2^117^
Amyloid to native	*Small molecules against the conversion to the native state*	*Stabilisation of the functional amyloid state*	*Currently not known*
Amyloid to droplet	*Small molecules promoting droplet hardening*	*Stabilisation of the functional amyloid state*	*Currently not known*

We anticipate that many approaches developed for the prevention of the conversion between the native and the amyloid states will be applicable to the conversion between the native and droplet states, as well as between the droplet and amyloid states. Since for diseases associated with the conversion of the amyloid state into the native or droplet state there are no currently known examples of therapeutic approaches, we suggested possible mechanisms of action (in italics).

Small molecules could be developed to modulate the interactions required for the stability of the droplet state. This is a mechanism of action that may for example be applicable to regulate cancer-driving super-enhancers^104^. Support for this type of approach is provided by the case of the steroidal alkaloid cyclopamine, which was shown to block the replication of the respiratory syncytial virus (RSV) by hardening the interactions within the condensates of the host proteins that drive viral RNA synthesis^45^. An appealing aspect of this strategy is that protein condensates can selectively partition small molecules. Mitoxantrone, for example, was observed to be selectively concentrated in nuclear condensates of the transcriptional coactivator MED1 and of nucleophosmin, driven by interactions of aromatic groups^105^. Similarly, small molecules can be used to shift the phase boundaries between the native and condensed states. The flavonoid compound myricetin was shown to inhibit droplet formation of the protein tau, resulting in decreased aggregation and toxicity^106^. The phase boundary of TDP-43 was modulated by multivalent interactions of an aromatic compound, bis-ANS^107^. Small molecules can be further used to specifically destabilise conformations that drive droplet formation, as in the case of allosteric inhibitors of the protein tyrosine phosphatase SHP2, which restored its normal MAPK activity^108^.

Small molecules can also be exploited to interfere with protein aggregation. The nucleation and elongation rates in the aggregation process of the Aβ peptide were inhibited by compounds that can be potentially developed as drugs for Alzheimer’s disease^109^. Small molecules may also stabilise the native conformations of aggregation-prone proteins, thus inhibiting the conversion between the native and amyloid states^110,111^. In addition, the inhibition of the formation of toxic oligomers and the removal of amyloid aggregates by degradation pathways and can be promoted by conformation-specific antibodies^112,113^.

Alternatively, activation of degradation pathways can be exploited for the removal of aberrant liquid-like condensates. The ubiquitination of Ras GTPase-activating protein-binding protein 1 (G3BP1) was shown to induce stress-granule disassembly via its interactions with the ubiquitin-dependent segregase valosin^114,115^. Valosin is known to activate autophagy, and its familial mutations lead to delayed droplet clearance^29^.

More generally, the modulation of the protein homoeostasis system can be explored for therapeutic purposes in protein condensation diseases. Molecular chaperone activation may stabilise aggregation-prone domains within droplets, as shown by the chaperoning the folded RNA-binding domain of FUS by the small heat shock protein HspB8, which inhibited the formation of aberrant condensates^116^. Cellular relocalisation may also prevent droplet aggregation, as shown by karyopherin-β2, which dissolves aberrant fibrillar hydrogels formed by FUS and hnRNPA1, and importin-α with karyopherin-β1 can revert TDP-43 aggregation^117^. Furthermore, as condensate assembly and biophysical properties are also regulated by the concentration of interaction partners^118^, modifying the expression level of these partners may offer a strategy to regulate the condensed states. For example, stress-granule hyper-assembly induced by medulloblastoma-associated DDX3 mutants can be reverted by depletion of other assembly factors^119^. In addition, one could activate or inhibit post-translational modifications that regulate the condensed states, such as those that stabilise the droplet state^120^, or promote formation of prion-like states^121^. Inhibitors of the dual-specificity kinase DYRK3 for example can prevent stress-granule dissolution^23^.

## Outlook

An increasing body of experimental observations suggests that protein condensation diseases may be ubiquitous. The strategies for drug discovery (Table 2) and the range of corresponding possible targets (Tables S1–S3) that we discussed here may be investigated further in future studies, given the growing interest in this therapeutic area. Although drug discovery targeting aberrant condensed states is likely to require different approaches than those used for stoichiometric complexes, proof-of-principle interventions to restore the balance between the different states of proteins have been already reported (Table 2). We anticipate that a better understanding of the nature of these diseases, and of the factors that regulate protein condensation, will promote the development of increasingly effective pharmacological approaches.

## Supplementary information

Supplementary Information

Description of Additional Supplementary Files

Supplementary Data 1

Supplementary Data 2

Supplementary Data 3

Supplementary data 4

## Source data

Source Data

## Data Availability

Gene–disease associations were derived from the DisGeNet database (http://disgenet.org), missense mutation-disease associations from the Human Variants Database (https://www.iitm.ac.in/bioinfo/huvarbase). Experimentally observed condensate-forming proteins were derived from three public databases: PhaSepDB data set (http://db.phasep.pro), PhaSePro (https://phasepro.elte.hu), LLPSDB (http://bio-comp.org.cn/llpsdb). For protein sequences, we used the UniProt database (uniprot.org). For GO enrichment we used the STRING (string-db.org) database. A list of protein condensation diseases is available at https://fuxreiterlab.github.io/databases_protein.html. The structures mentioned in this work are publicly available under the PDB accession codes 2N3X (Solution Structure of TDP-43 Amyloidogenic Core Region) and 7KWZ (TDP-43 LCD amyloid fibrils). Source data are provided with this paper.
