# Supplementary material for: Protein condensation diseases: therapeutic opportunities
Source: Nat Commun. 2022 Sep 22;13:5550. doi: 10.1038/s41467-022-32940-7 (PMC9500012; doi:10.1038/s41467-022-32940-7)
Supplement: Supplementary file 1 — Supplementary Information [file 41467_2022_32940_MOESM1_ESM.pdf]

Supplementary Information

Supplementary Methods

Widespread association of human diseases with aberrant protein condensation

We analysed 4433 human proteins reported to undergo liquid-liquid phase separation or observed as components in membraneless organelles (MLO, **Supplementary data set: Table S1**). In addition, we analysed 5757 human proteins predicted to drive droplet formation[1] (PC, predicted condensates) (**Supplementary data set: Table S1**). We currently have no experimental evidence or predictions that other 10635 human proteins (UniProt, May 2021) can undergo condensate formation. Although we will refer to this class as non-condensate-forming proteins (NONE), many of them comprise regions that could in principle promote their partition into droplets[1].

Our analysis indicates that over 3000 of the 9277 diseases from curated resources in the DisGeNet database (http://disgenet.org)[2] (UniProt, Comparative Toxicogenomics Database, Orphanet, Clinical Genome Resource, Genomics England PanelApp, Cancer Genome Interpreter and Psychiatric disorders Gene association Network), and over 4500 of the 21552 diseases from all resources also including Human Phenotype Onthology, ClinVar and genome-wide association studies NHGRI-EBI-GWAS) are associated with genes that all encode condensate-forming proteins (**Supplementary data set: Table S2**). In addition, based on the current knowledge, droplet-associated genes can be associated with about three quarters of the over 20 thousand diseases in the DisGeNet database.

Furthermore, we analyzed 644,521 disease-associated missense variants in human proteins in the Human Variants Database[3], finding that 145,924 missense mutations in experimentally-identified condensate components and 232,866 variants in predicted droplet-forming proteins are associated with human diseases. Since about one third of these mutations are in regions predicted to drive droplet formation[1], we estimate that over 100,000 disease-associated missense mutations may alter condensate properties, shift the phase boundary, or promote aggregation. In addition, for 327 proteins forming membraneless organelles and for nearly 800 predicted droplet-forming proteins, over 70% of disease-associated missense mutations are in regions that likely drive condensate formation (**Supplementary data set: Table S3**). The accumulation of missense mutations in droplet-promoting regions suggests that disease-causing mutations alter condensate properties.

Datasets of droplet-forming proteins

According to available experimental evidence, we listed the droplet-forming human proteins that are components of membraneless-organelles (MLO). These proteins were either observed to undergo spontaneous liquid-liquid phase separation, as droplet-driver proteins, or were identified by high-throughput studies as components of cellular condensates (droplet-clients). Droplet-driver proteins were collected from public databases (PhaSepDB dataset (http:/db.phasep.pro)[4], PhaSePro (https://phasepro.elte.hu)[5], LLPSDB (http://bio-comp.org.cn/llpsdb)[6]) in a previous study[1], and complemented by new cases in the updated PhaSepDB v2[4]. Proteins classified as 'PS-SELF' were defined as droplet drivers. Proteins

classified as 'PS-OTHER' and components of membraneless organelles identified by high-throughput studies by organelle purification[7,8], affinity purification[9,10], immunofluorescence image based screen[11,12], and proximity labelling[13,14] were assembled as droplet-clients. The human MLO dataset contained 4434 proteins, 462 droplet-drivers and 3972 droplet-clients (**Supplementary data set: Table S1**). We note that most droplet-forming proteins are also amyloid-forming proteins[15,16].

As more proteins may be expected to drive phase separation than those currently deposited in public datasets, we also assembled proteins predicted to undergo liquid-liquid phase separation using FuzDrop ($p_{LPS} \geq 0.60$)[1]. In addition to the experimentally-identified condensate-forming proteins, we assembled 5757 predicted human droplet-driver proteins (PC: predicted condensates, **Supplementary data set: Table S1**), using UniProt (May 2021).

Datasets of non-condensate proteins

10635 proteins in UniProt (May 2021) that have not been experimentally observed yet to undergo liquid-liquid phase separation, neither identified as components of membraneless organelles, nor predicted to drive condensate-formation were considered as non-condensate forming proteins. These proteins may also have droplet-promoting regions that can facilitate their partitioning into condensates.

Disease-gene associations

Disease-gene associations were derived from the DisGeNet database (http://disgenet.org)[2]. We analyzed 9277 diseases associated with protein-coding genes derived from curated resources (UniProt, Comparative Toxicogenomics Database, Orphanet, Clinical Genome Resource, Genomics England PanelApp, Cancer Genome Interpreter and Psychiatric disorders Gene association Network) and 21552 diseases associated with protein-coding genes also including inferred data (Human Phenotype Onthology, ClinVar and genome-wide association studies NHGRI-EBI-GWAS). We only analyzed pathologies defined as disease, and not phenotypes or groups. Diseases were termed as in the database using MSH classifications.

Missense variants affecting protein condensates

We analyzed 644,521 missense mutations of 17450 human proteins in the Human Variants Database**,** which was assembled mutational data from 1000 Genomes, ClinVar, COSMIC, SwissVar, and Humsavar. Disease names in HuVarBAse were termed based on Genetic Testing Registry (https://www.ncbi.nlm.nih.gov/gtr/) and we considered all the disease associations of the missense variants. We grouped missense variants based on the condensate-forming ability of the corresponding proteins, whether they were identified as components of membraneless organelles (MLO), or were predicted to form condensates (PC) or belonged to proteins not known to form condensates (NONE).

Ranking of protein condensation diseases

To identify diseases with major contributions from mutations affecting condensate properties, we used two approaches. In the first, based on disease-gene associations in the DisGeNet database, we collected all the protein-coding genes associated with a given disease. We then

determined the contributions of genes that encoded experimentally observed droplet-forming proteins (MLO), and genes encoded predicted condensates (PC) by calculating the fraction of genes encoding droplet-forming proteins (f_{DROP}=(n_{MLO} + n_{PC})/n_{TOT}). We ranked the diseases based on the fraction of genes encoding droplet-forming proteins forming membraneless-organelles (**Supplementary data set: Table S2**). This ranking evaluated the contribution of genes encoding condensate-forming and non-condensate forming proteins and could identify those diseases, where the associated droplet-coding genes make major contributions (**Supplementary data set: Table S2**). Diseases were classified according to the standard classification system (MSH as defined in the DisGeNet database).

In the second approach, we determined the number of missense variants of proteins that form membraneless organelles (n_{MLO}), the number of missense variants of proteins that are predicted to form condensates (n_{PC}) and missense variants of proteins that are not known to form condensates (n_{NONE}). Then we computed the fraction of missense mutations in droplet-forming proteins (f_{DROP}=(n_{MLO} + n_{PC})/n_{TOT}). We ranked the diseases based on the fraction of missense mutations in membraneless component proteins and predicted condensate-forming proteins. In this analysis we only ranked diseases, which are associated with proteins, where most missense mutations fall into droplet-promoting regions (**Supplementary data set: Table S3**).

Pathways associated with protein condensation diseases

We analyzed the biochemical pathways enriched in disease-associated droplet-forming proteins. In particular, we computed through the STRING database search tools[17] the enrichment of biological processes and molecular functions of the Gene Ontology database, KEGG pathways and Wikipath, which are enriched in disease-associated genes (**Supplementary data set: Table S2**), encoding membraneless-organelle forming proteins and predicted condensates (**Supplementary data set: Table S4**).

References

1 Hardenberg, M., Horvath, A., Ambrus, V., Fuxreiter, M. & Vendruscolo, M. Widespread occurrence of the droplet state of proteins in the human proteome. *Proc. Natl. Acad. Sci. USA* **117**, 33254-33262 (2020).

2 Piñero, J. *et al.* The disgenet knowledge platform for disease genomics: 2019 update. *Nucl. Acids. Res.* **48**, D845-D855 (2020).

3 Ganesan, K., Kulandaisamy, A., Binny Priya, S. & Gromiha, M. M. Huvarbase: A human variant database with comprehensive information at gene and protein levels. *PLoS One* **14**, e0210475 (2019).

4 You, K. *et al.* Phasepdb: A database of liquid–liquid phase separation related proteins. *Nucl. Acids. Res.* **48**, D354-D359 (2020).

5 Mészáros, B. *et al.* Phasepro: The database of proteins driving liquid–liquid phase separation. *Nucl. Acids. Res.* **48**, D360-D367 (2020).

6 Wang, X. *et al.* Llpsdb v2. 0: An updated database of proteins undergoing liquid–liquid phase separation in vitro. *Bioinformatics* **38**, 2010-2014 (2022).

7 Hubstenberger, A. *et al.* P-body purification reveals the condensation of repressed mrna regulons. *Mol. Cell* **68**, 144-157. e145 (2017).

8 Andersen, J. S. *et al.* Nucleolar proteome dynamics. *Nature* **433**, 77-83 (2005).

9 Ayache, J. *et al.* P-body assembly requires ddx6 repression complexes rather than decay or ataxin2/2l complexes. *Mol. Biol. Cell* **26**, 2579-2595 (2015).

10 Jonson, L. *et al.* Molecular composition of imp1 ribonucleoprotein granules. *Mol. Cell Proteomics* **6**, 798-811 (2007).

11 Fong, K.-w. *et al.* Whole-genome screening identifies proteins localized to distinct nuclear bodies. *J. Cell Biol.* **203**, 149-164 (2013).

12 Berchtold, D., Battich, N. & Pelkmans, L. A systems-level study reveals regulators of membrane-less organelles in human cells. *Mol. Cell* **72**, 1035-1049. e1035 (2018).

13 Markmiller, S. *et al.* Context-dependent and disease-specific diversity in protein interactions within stress granules. *Cell* **172**, 590-604. e513 (2018).

14 Youn, J.-Y. *et al.* High-density proximity mapping reveals the subcellular organization of mrna-associated granules and bodies. *Mol. Cell* **69**, 517-532. e511 (2018).

15 Vecchi, G. *et al.* Proteome-wide observation of the phenomenon of life on the edge of solubility. *Proc. Natl. Acad. Sci. USA* **117**, 1015-1020 (2020).

16 Vendruscolo, M. & Fuxreiter, M. Sequence determinants of the aggregation of proteins within condensates generated by liquid-liquid phase separation. *J. Mol. Biol.* **434**, 167201 (2022).

17 Szklarczyk, D. *et al.* The string database in 2021: Customizable protein–protein networks, and functional characterization of user-uploaded gene/measurement sets. *Nucl. Acids. Res.* **49**, D605-D612 (2021).

18 The UniProt Consortium. UniProt: The universal protein knowledgebase in 2021. *Nucl. Acids. Res.* **49**, D480-D489 (2021).
