# Supplementary material for: Protein condensation diseases: therapeutic opportunities
Source: Nat Commun. 2022 Sep 22;13:5550. doi: 10.1038/s41467-022-32940-7 (PMC9500012; doi:10.1038/s41467-022-32940-7)
Supplement: Supplementary file 2 — Description of Additional Supplementary Files [file 41467_2022_32940_MOESM2_ESM.docx]

**Description of Additional Supplementary Files**

**Supplementary Table S1. List of human proteins associated with liquid-liquid phase separation.**

**Sheet 1: List of condensate-forming human proteins based on experimental evidence (MLOs) or predictions (PC).**

Droplet-driver proteins are observed to undergo spontaneous liquid-liquid phase separation. Droplet-client proteins are identified as recruited as components of cellular condensates (MLO). This list also includes 92 alternatively spliced protein isoforms observed to undergo liquid-liquid phase separation. Condensate-forming proteins from predictions (PC) have a high FuzDrop score (p_LLPS_ score ≥ 0.6)^1^; these proteins are predicted to be able to undergo spontaneous liquid-liquid phase separation and thus can play roles as droplet-drivers.

**Sheet 2: Biological functions of condensate-forming proteins (MLOs).**

Gene ontology of droplet-driver and droplet-client human proteins, including biological processes, molecular functions and cellular components. Functions associated with MLO proteins in the UniProt database are also displayed^18^.

**Sheet 3: Biological functions of predicted condensate-forming proteins (PC).**

Gene ontology, including biological processes, molecular functions and cellular components, of human proteins predicted to spontaneously undergo liquid-liquid phase separation. Functions associated with these proteins in the UniProt database are also displayed^18^.

**Supplementary Table S2. Ranking of human diseases based on their links with droplet-forming proteins.**

**Sheet 1: Curated data.** The 5803 diseases in the table are taken from the 9277 diseases in curated resources in the DisGeNet database, and ranked by the number of genes encoding droplet-forming proteins (*N(DROP)*, see Legend). The sheet lists the disease-associated genes and corresponding encoded proteins that are components of membraneless organelles (MLO) and proteins predicted to form condensate (PC).

**Sheet 2: All data.** The 16393 diseases in the table are taken from the 21552 diseases in all resources in the DisGeNet database, and ranked by the number of genes encoding droplet-forming proteins (*N(DROP)*, see Legend).

**Sheet 3: Orphan diseases**. A list of rare (orphan) diseases with at least one third of the contributing genes associated with protein condensation.

**Supplementary Table S3. Gene-disease associations based on missense mutations.**

**Sheet 1: Missense mutations in droplet-promoting regions (DPRs).**  We analyzed 644,521 disease-associated missense mutations of 17450 human proteins in the Human Variants Database (HuVarBase)^3^, for their position in the protein sequence. For each protein, we computed the fraction of missense mutations in droplet-promoting regions (*N_mut_*(DRP)/*N_mut_*(tot)). We thus identified proteins for which over 70% of the missense mutations are associated with droplet-promoting regions (DPRs) in condensate-forming proteins (*f(m,dpr)*, see Legend).

**Sheet 2: Condensation-related diseases in HuVarBase.** Diseases associated with proteins for which over 70% of missense mutations are in droplet-promoting regions (DPRs). Diseases were ranked by the fraction of disease-associated missense mutations of droplet-forming proteins as compared to those of non-condensate forming proteins (*Nmut(D)/Nmut(tot)*, see Legend). This ranking evaluates the contribution of missense mutations of experimental (MLO) and predicted (PC) droplet-forming proteins to the disease by comparing it to those of non-condensate forming proteins.

**Supplementary Table S4. Biological pathways enriched in condensate-forming disease-associated proteins (from Table S2).**

**Sheet 1: GO process MLO.** Biological processes (GO) enriched in experimentally identified droplet-forming proteins (MLO) versus the human proteome.

**Sheet 2: GO molecular function MLO:** Molecular function (GO) enriched in experimentally identified droplet-forming proteins versus the human proteome.

**Sheet 3: KEGG pathways MLO:** KEGG pathways enriched in experimentally identified droplet-forming proteins (MLO) versus the human proteome.

**Sheet 4: Wikipath pathways MLO.** Wikipath pathways enriched in experimentally identified droplet-forming proteins (MLO) versus the human proteome.

**Sheet 5: GO process PC.** Biological processes (GO) enriched in predicted droplet-forming proteins (PC) versus the human proteome

**Sheet 6: GO molecular function PC.** Molecular function (GO) enriched in predicted droplet-forming proteins (PC) versus the human proteome.

**Sheet 7: KEGG pathways PC.** KEGG pathways enriched in predicted droplet-forming proteins (PC) versus the human proteome.

**Sheet 8: Wikipath pathways PC.** Wikipath pathways enriched in predicted droplet-forming proteins (PC) versus the human proteome.
